# Supplementary material for: Unravelling reference bias in ancient DNA datasets
Source: Bioinformatics. 2024 Jul 3;40(7):btae436. doi: 10.1093/bioinformatics/btae436 (PMC11254355; doi:10.1093/bioinformatics/btae436)
Supplement: btae436_Supplementary_Data [file btae436_supplementary_data.zip › S1-S4_data_plots.zip!/S1-S4_data/rhino_sim_plots.zip!/rhino_sim_plots/bwaaln_rhino_div3_10end_by_MQ.pdf]

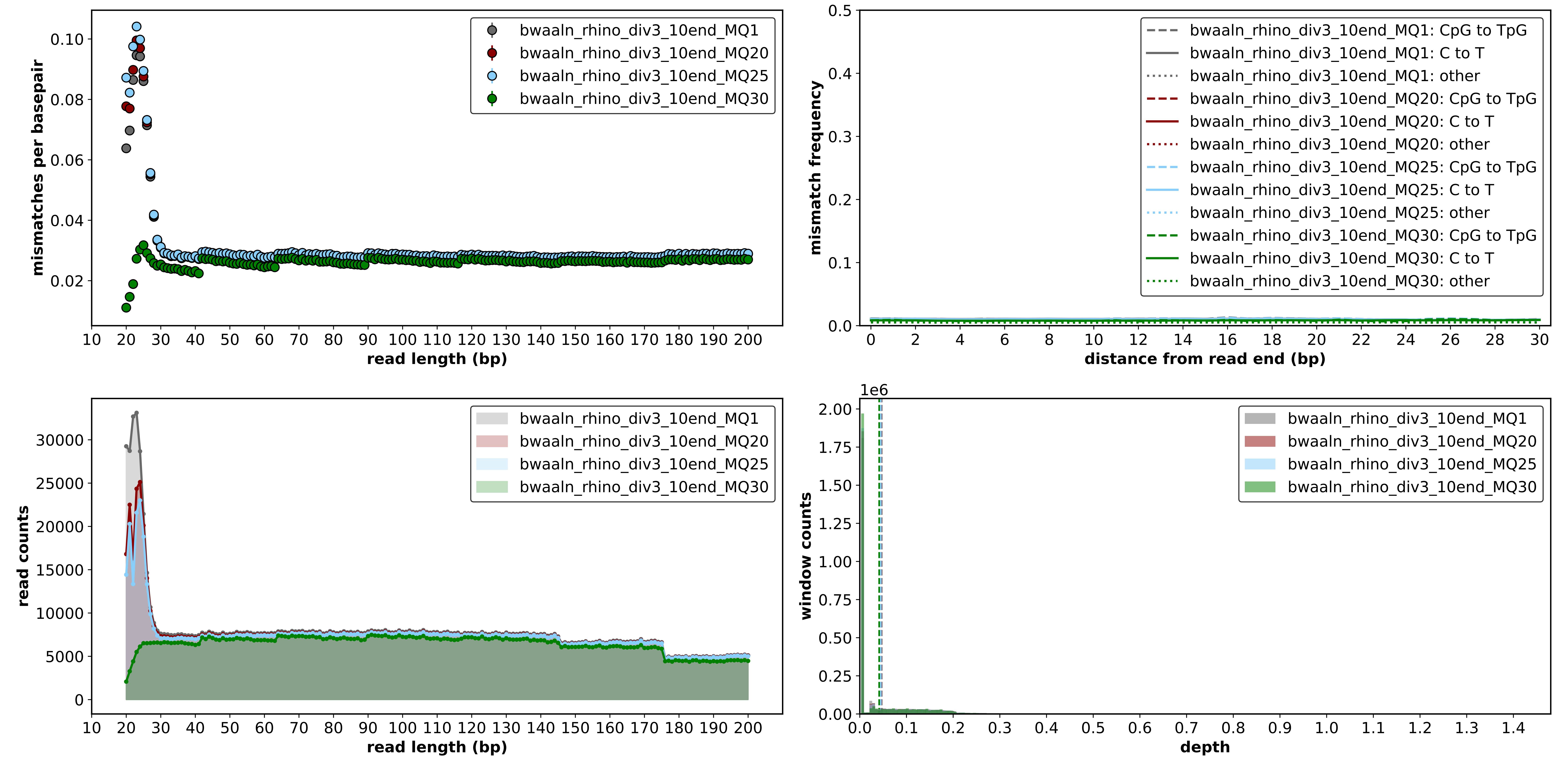
mismatches per basepair
read length (bp)
bwaaln_rhino_div3_10end_MQ1
bwaaln_rhino_div3_10end_MQ20
bwaaln_rhino_div3_10end_MQ25
bwaaln_rhino_div3_10end_MQ30
0.02
0.04
0.06
0.08
0.10
10 20 30 40 50 60 70 80 90 100 110 120 130 140 150 160 170 180 190 200
mismatch frequency
distance from read end (bp)
0.0
0.1
0.2
0.3
0.4
0.5
0 2 4 6 8 10 12 14 16 18 20 22 24 26 28 30
bwaaln_rhino_div3_10end_MQ1: CpG to TpG
bwaaln_rhino_div3_10end_MQ1: C to T
bwaaln_rhino_div3_10end_MQ1: other
bwaaln_rhino_div3_10end_MQ20: CpG to TpG
bwaaln_rhino_div3_10end_MQ20: C to T
bwaaln_rhino_div3_10end_MQ20: other
bwaaln_rhino_div3_10end_MQ25: CpG to TpG
bwaaln_rhino_div3_10end_MQ25: C to T
bwaaln_rhino_div3_10end_MQ25: other
bwaaln_rhino_div3_10end_MQ30: CpG to TpG
bwaaln_rhino_div3_10end_MQ30: C to T
bwaaln_rhino_div3_10end_MQ30: other
read counts
read length (bp)
0
5000
10000
15000
20000
25000
30000
10 20 30 40 50 60 70 80 90 100 110 120 130 140 150 160 170 180 190 200
bwaaln_rhino_div3_10end_MQ1
bwaaln_rhino_div3_10end_MQ20
bwaaln_rhino_div3_10end_MQ25
bwaaln_rhino_div3_10end_MQ30
window counts
depth
1e6
0.00
0.25
0.50
0.75
1.00
1.25
1.50
1.75
2.00
0.0 0.1 0.2 0.3 0.4 0.5 0.6 0.7 0.8 0.9 1.0 1.1 1.2 1.3 1.4
bwaaln_rhino_div3_10end_MQ1
bwaaln_rhino_div3_10end_MQ20
bwaaln_rhino_div3_10end_MQ25
bwaaln_rhino_div3_10end_MQ30
